# Accidental Poisoning with Bad Calomel in France

**Published:** 1849-01

**Authors:** 


*Accidental poisoning with bad Calomel in France.*—Calomel is much used in this country, and we are happy to find that the absence of accidents caused by this salt evidently point to the care with which the bi-chloride is separated from it. A gentlemen has just fallen a victim in France to the faulty preparation of calomel. His physician ordered twelve grains of the salt (prepared by steam) to be suspended in a gummy emulsion. One spoonful was to be taken every hour. The first spoonful produced an alvine evacuation, the second, dijections of large quantities of mucus, then followed a frightful convulsive fit; a third spoonful confirmed the convulsions, and the next day, at 12 o'clock the patient was dead. The whole mixture was immediately analyzed; it contained no less than from three to four grains of bi-chloride of mercury. Fifteen grains of the calomel taken from the establishment of the chemist where the mixture had been prepared gave the same relative results. A very simple way of testing calomel, among the many which are in use, is to place a little on a well scoured sheet of copper, and then treat it with ether. On rubbing the metal slightly on the point where the evaporation is taking place, a brilliant amalgram is formed. This is enough to show that the calomel contains a soluble salt of mercury, that it is poisonous, and ought to be rejected. *Ibid.*

